# Solid Dynamic Models for Analysis of Stress and Strain in Human Hearts

**DOI:** 10.1155/2012/634534

**Published:** 2012-02-12

**Authors:** Qiu Guan, Miaomiao Lu, Xiaoyan Wang, Cao Jiang

**Affiliations:** ^1^College of Computer Science and Technology, Zhejiang University of Technology, Hangzhou 310023, China; ^2^College of Information Engineering, Zhejiang University of Technology, Hangzhou 310023, China

## Abstract

This paper proposes a solid model based on four-dimensional trivariate B-spline for strain and stress analysis of ventricular myocardium. With a series of processing steps in the four-dimensional medical images, the feature points of ventricular inner and outer wall are obtained. A B-spline surface is then used to build the dynamic deformation model of the myocardial walls. With such a surface model, a hexahedron control mesh can be constructed by sweeping the cloud data, and the ventricular solid model is built by fitting the trivariate B-spline parameters. Based on these models, a method of isogeometric analysis can be applied to calculate the stress and strain continuously distributed in the ventricle. The model is represented smoothly in the cylindrical coordinate system and is easy to measure myocardium dynamics for finding abnormal motion. Experiments are carried out for comparing the stress and strain distribution. It is found that the solid model can determine ventricular dynamics which can well reflect the deformation distribution in the heart and imply early clues of cardiac diseases.

## 1. Introduction

Cardiovascular diseases are currently the leading cause of death in the world, and the rate of death is increasing each year in many countries. Therefore, more and more physiologists and researchers make efforts to understand how the heart works and how to diagnosis and treat the heart diseases. Dynamics and kinetics of the left cardiac ventricle are the primary representation of the cardiac motion, as a series of systolic and diastolic motions of the left cardiac ventricle make the heart pump blood to circulate the whole body. Furthermore, the stress and strain express the characteristics of elasticity and motion of myocardial walls [1].

**Figure 1:**
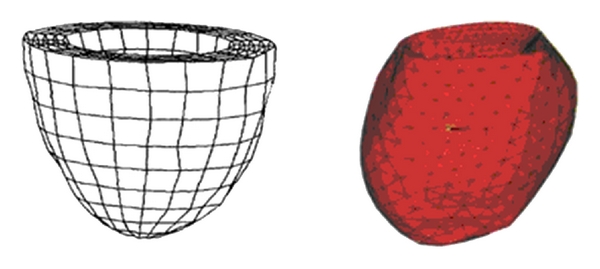
Finite element models of left ventricle in the literature [2, 3].

With assist of computer medical imaging technology, such as magnetic resonance imaging (MRI), computed tomography (CT), positron emission tomography, single photon emission computed tomography, ultrasound, and X-ray, these imaging techniques can give different representation of the heart. In this paper, the endocardium and epicardium shapes of a ventricle are constructed with a segmentation technique in MRI images. For constructing model, finite element (FE) models are the most commonly used in biomechanics. Especially, hexahedral and tetrahedral FE models are popular in the representation of cardiac model, for example, Figure 1 [2, 3]. Comparing with the FE model, left ventricular CAD (computer-aided design) model is more representative of the true ventricle in the shape, such as B-spline surface model. Its continuity and smoothness are better than the FE model. However, it is difficult to apply FE analysis directly to the CAD model [4].

The appearance of isogeometric analysis makes it possible to carry out mechanical analysis directly by a CAD model. As a new computational technique, isogeometric analysis improves on and generalizes the standard FE method and has been proven to be a powerful method exceeding the FE method [5]. Inspired by isogeometric analysis, this paper constructs a solid model based on trivariate B-spline and calculates the strain and stress of a ventricle by this model. Finally, it shows the distribution of the strain and stress on the solid model.

## 2. Model Representation

### 2.1. B-Spline Curves and Surfaces

One can get a B-spline curve of order *k*. By [6]

(1) $$\begin{array}{cc} C\left(t\right)=\overset{n}{\underset{i=0}{\sum }}p_{i}{\;N}_{i,k}\;\left(t\right), & \;t_{k}\leq t< \end{array}t_{k+1},$$

where *p*_0_, *p*_1_,…, *p*_*n*_ are the control points of *C*(*t*), the linear interpolation of control points is called control polygon. *N*_*i*,*k*_(*u*) is B-spline basis function of degree *k*, which is determined by a group of nondecreasing normalized sequence *T*: *t*_0_ ≤ *t*_1_ ≤ ⋯≤*t*_*n*+*k*+1_. The sequence can be determined by Riesenfeld method and Hartley-Judd method [7].

The definition of *N*_*i*,*k*_(*u*) can be expressed as

(2) $$\;\begin{array}{c} N_{i,0}\left(t\right)=\{\begin{array}{cc} 1, & \text{if}\;t_{i}\leq t<t_{i+1,}\\ 0, & \text{else} \end{array}\\ N_{i,k}\left(t\right)=\frac{t-t_{i}}{t_{i+k}-t_{i}}N_{i,k-1}\left(t\right)+\frac{t_{i+k+1}-t}{t_{i+k+1}-t_{i+1}}N_{i+1,k-1}\left(t\right)\\ \text{define}\;\frac{0}{0}=0. \end{array},$$

With a tensor product, given a (*n* + 1)×(*m* + 1) control mesh *p*_*i*,*j*_ and knot vector *T*: *t*_0_ ≤ *t*_1_ ≤ ⋯≤*t*_*n*+*k*+1_, a B-spline surface of degree *k*_1_ in the *t* direction and degree *k*_2_ in the *w* direction is defined as

(3) $$S\left(t,w\right)=\overset{n}{\underset{i=0}{\sum }}\overset{m}{\underset{\;j=0}{\sum }}p_{i,j}N_{i,k_{1}}\left(t\right)N_{j,k_{2}}\left(w\right),$$

where *S* is a point on the defined surface, and *t* and *w* are usually representing longitude and latitude; respectively, *N*_*i*,*k*_1__(*t*) and *N*_*i*,*k*_1__(*w*) are the basis functions in the *t* and *w* directions with a degree of *k*_1_ and *k*_2_.

### 2.2. Volumetric B-Splines

A volumetric B-spline can also be defined by

(4) $$V\left(t,w,u\right)=\overset{n}{\underset{i=0}{\sum }}\overset{m}{\underset{\;j=0}{\sum }}\overset{l}{\underset{k}{\sum }}p_{i,j,l}N_{i,k_{1}}\left(t\right)N_{j,k_{2}}\left(w\right)N_{k,k_{3}}\left(u\right).$$

Comparing with the model in Cartesian coordinates, a cylindrical coordinate model is better approximate to the shape of the heart [8]. Volumetric B-spline is the extension of B-spline surface, which can be given by the following two steps in cylindrical coordinates.

(1) Coordinate transform. A (n + 1)×(m + 1)×(l + 1) control mesh *p*_*i*,*j*,*l*_ with the points (*x*_*ijk*_, *y*_*ijk*_, *z*_*ijk*_) in Cartesian coordinate is transformed into cylindrical coordinate by

(5) $$\begin{array}{c} r_{ijk}=\sqrt{x_{ijk}^{2}+y_{ijk}^{2}},\\ \theta_{ijk}=\{\begin{array}{cc} \pi /2 & \text{if}{\;x}_{ijk}=0,y_{ijk}>0,\\ 3\pi /2 & \text{if}\;x_{ijk}=0,y_{ijk}<0,\\ a\;\tan\left(\frac{y_{ijk}}{x_{ijk}}\right) & \text{if}\;x_{ijk}>0,y_{ijk}\geq 0,\\ \pi +a\;\tan\left(\frac{y_{ijk}}{x_{ijk}}\right) & \text{if}\;x_{ijk}<0,\\ 2\pi +a\;\tan\left(\frac{y_{ijk}}{x_{ijk}}\right) & \text{if}\;x_{ijk}>0,y_{ijk}<0, \end{array}\\ z_{ijk}=z_{ijk}. \end{array}$$

(2) The representation of volumetric B-spline in cylindrical coordinate is

(6) $$\begin{array}{c} r\left(t,w,u\right)=\overset{n}{\underset{i=0}{\sum }}\overset{m}{\underset{j=0}{\sum }}\overset{l}{\underset{k}{\sum }}r_{i,j,k}N_{i,k_{1}}\left(t\right)N_{j,k_{2}}\left(w\right)N_{k,k_{3}}\left(u\right),\\ \theta \left(t,w,u\right)=\overset{n}{\underset{i=0}{\sum }}\overset{m}{\underset{j=0}{\sum }}\overset{l}{\underset{k}{\sum }}\theta_{i,j,k}N_{i,k_{1}}\left(t\right)N_{j,k_{2}}\left(w\right)N_{k,k_{3}}\left(u\right),\\ z\left(t,w,u\right)=\overset{n}{\underset{i=0}{\sum }}\overset{m}{\underset{j=0}{\sum }}\overset{l}{\underset{k}{\sum }}z_{i,j,k}N_{i,k_{1}}\left(t\right)N_{j,k_{2}}\left(w\right)N_{k,k_{3}}\left(u\right). \end{array}$$

## 3. Determination of Stress and Strain

### 3.1. Elasticity

**Figure 2:**
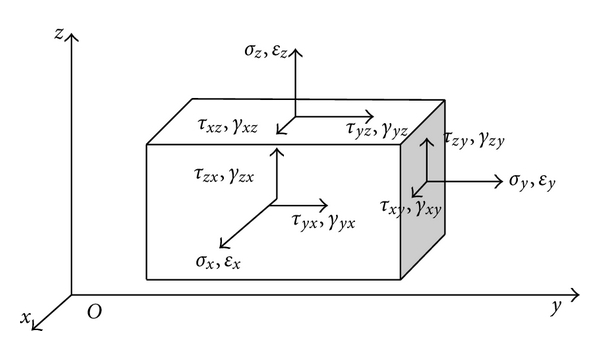
Three-dimensional stress and strain components.

Stress and strain actually reflect elasticity of ventricular myocardial walls [9]. Calculations of the stress and strain can be inspired from the idea of elasticity theory. In 3D space, set *u*, *v*, *w* as the displacements in *x*, *y*, *z* direction. Strain vector and stress vector are *σ* = [*σ*_*x*_, *σ*_*y*_, *σ*_*z*_, *τ*_*xy*_, *τ*_*yz*_, *τ*_*xz*_]^*T*^ and *ε* = [*ε*_*x*_, *ε*_*y*_, *ε*_*z*_, *γ*_*xy*_, *γ*_*yz*_, *γ*_*xz*_]^*T*^, respectively, as shown in Figure 2.

The relation between strain and displacement is

(7) $$\varepsilon =\{\begin{array}{c} \varepsilon_{x}=\frac{\partial u}{\partial x},\\ \varepsilon_{y}=\frac{\partial v}{\partial y},\\ \varepsilon_{z}=\frac{\partial w}{\partial z},\\ \gamma_{xy}=\frac{\partial u}{\partial y}+\frac{\partial v}{\partial x},\\ \gamma_{yz}=\frac{\partial v}{\partial z}+\frac{\partial w}{\partial y},\\ \gamma_{xz}=\frac{\partial u}{\partial z}+\frac{\partial w}{\partial x}. \end{array}$$

Physical relationship or the relation between strain and stress is

(8) σ=Dε,

where *D* is the elastic matrix defined as

(9) $$\;\begin{array}{cc} D & =\frac{E\left(1-\mu \right)}{\left(1+\mu \right)\left(1-2\;\mu \right)}\\ & \;\times \left[\begin{array}{cccc} 1 & \frac{\mu }{1-\mu } & \frac{\mu }{1-\mu } & \begin{array}{ccc} 0 & 0 & 0 \end{array}\\ \frac{\mu }{1-\mu } & 1 & \frac{\mu }{1-\mu } & \begin{array}{ccc} 0 & 0 & 0 \end{array}\\ \frac{\mu }{1-\mu } & \frac{\mu }{1-\mu } & 1 & \begin{array}{ccc} 0 & 0 & 0 \end{array}\\ \begin{array}{c} 0\\ 0\\ 0 \end{array} & \begin{array}{c} 0\\ 0\\ 0 \end{array} & \begin{array}{c} 0\\ 0\\ 0 \end{array} & \begin{array}{ccc} \begin{array}{c} H\\ 0\\ 0 \end{array} & \begin{array}{c} 0\\ H\\ 0 \end{array} & \begin{array}{c} 0\\ 0\\ H \end{array} \end{array} \end{array}\right] \end{array},$$

where *E* is elasticity modulus, *μ* is Poisson's ratio, and *H* = (1 − 2 *μ*)/2(1 − *μ*). With (9), strain and stress can be determined.

### 3.2. Isogeometric Analysis

We regard B-spline basis functions as the displacement function and also the basis function of the ventricular shape, to calculate the stress and strain. Solid models at adjacent time points can be reconstructed by (4) with corresponding control points. The continuous displacements of the solid model correspond the displacements of control points, that is,

(10) $$\text{Disp}\left(t_{c},t_{l},t_{r}\right)=\overset{n}{\underset{i=0}{\sum }}\overset{m}{\underset{\;j=0}{\sum }}\overset{l}{\underset{\;k=0}{\sum }}d_{i,j,l}N_{i,k_{1}}\left(t_{c}\right)N_{j,k_{2}}\left(t_{l}\right)N_{k,k_{3}}\left(t_{r}\right),$$

where *d*_*i*,*j*,*l*_ is the displacements of control points of Disp(*t*_*c*_, *t*_*l*_, *t*_*r*_). *n*, *m*, *l* define the control net, *t*_*c*_, *t*_*l*_, and *t*_*r*_ are the knot vectors in the directions of circumference, long axis and radius, respectively. For *X*, *Y*, *Z* directions, the displacements *DX*, *DY*, *DZ* are defined as

(11) $$\begin{array}{c} DX\left(t_{c},t_{l},t_{r}\right)=\overset{n}{\underset{i=0}{\sum }}\overset{m}{\underset{j=0}{\sum }}\overset{l}{\underset{\;k=0}{\sum }}{d^{x}}_{i,j,l}N_{i,k_{1}}\left(t_{c}\right)N_{j,k_{2}}\left(t_{l}\right)N_{k,k_{3}}\left(t_{r}\right),\\ DY\left(t_{c},t_{l},t_{r}\right)=\overset{n}{\underset{i=0}{\sum }}\overset{m}{\underset{j=0}{\sum }}\overset{l}{\underset{\;k=0}{\sum }}{d^{y}}_{i,j,l}N_{i,k_{1}}\left(t_{c}\right)N_{j,k_{2}}\left(t_{l}\right)N_{k,k_{3}}\left(t_{r}\right),\\ DZ\left(t_{c},t_{l},t_{r}\right)=\overset{n}{\underset{i=0}{\sum }}\overset{m}{\underset{j=0}{\sum }}\overset{l}{\underset{\;k=0}{\sum }}{d^{z}}_{i,j,l}N_{i,k_{1}}\left(t_{c}\right)N_{j,k_{2}}\left(t_{l}\right)N_{k,k_{3}}\left(t_{r}\right), \end{array}$$

where *d*_*xi*,*j*,*l*_, *d*_*yi*,*j*,*l*_, *d*_*zi*,*j*,*l*_ are the displacements of control points in *X*, *Y*, *Z* directions.

By elasticity theory, the strain of one point in the model can be calculated

(12) $$\begin{array}{c} \varepsilon_{x}=\frac{\partial u}{\partial x}=\frac{\partial DX}{\partial x}=\frac{\partial N}{\partial x}d^{x},\\ \varepsilon_{y}=\frac{\partial v}{\partial y}=\frac{\partial DY}{\partial y}=\frac{\partial N}{\partial y}d^{y},\\ \varepsilon_{z}=\frac{\partial w}{\partial z}=\frac{\partial DZ}{\partial z}=\frac{\partial N}{\partial z}d^{z},\\ \gamma_{xy}=\frac{\partial u}{\partial y}+\frac{\partial v}{\partial x}=\frac{\partial DX}{\partial y}+\frac{\partial DY}{\partial x}=\frac{\partial N}{\partial y}d^{x}+\frac{\partial N}{\partial x}d^{y},\\ \gamma_{yz}=\frac{\partial v}{\partial z}+\frac{\partial w}{\partial y}=\frac{\partial DY}{\partial z}+\frac{\partial DZ}{\partial y}=\frac{\partial N}{\partial z}d^{y}+\frac{\partial N}{\partial y}d^{z},\\ \gamma_{xz}=\frac{\partial u}{\partial z}+\frac{\partial w}{\partial x}=\frac{\partial DX}{\partial z}+\frac{\partial DZ}{\partial x}=\frac{\partial N}{\partial z}d^{x}+\frac{\partial N}{\partial x}d^{z}, \end{array}$$

where *N* = *N*_*i*,*k*_1__(*t*_*c*_)*N*_*j*,*k*_2__(*t*_*l*_)*N*_*k*,*k*_3__(*t*_*r*_) is the B-spline basis function, and *dx*, *dy*, and *dz* are displacement values of the corresponding points.

Then the derivatives of B-spline basis functions in *X*, *Y*, *Z* directions can be derived in each parameter direction

(13) $$\;\begin{array}{cc} \frac{\partial N}{\partial t_{c}} & =\left(k_{1}-1\right)\left[\frac{N_{i,k_{1}-1}\left(t_{c}\right)}{t_{c,i+k_{1}-1}-t_{c,i}}-\frac{N_{i+1,k_{1}-1}\left(t_{c}\right)}{t_{c,i+k_{1}}-t_{c,i+1}}\right]\\ & \;\times N_{j,k_{2}}\left(t_{l}\right)N_{k,k_{3}}\left(t_{r}\right),\\ \frac{\partial N}{\partial t_{l}} & =\left(k_{2}-1\right)\left[\frac{N_{j,k_{2}-1}\left(t_{l}\right)}{t_{l,j+k_{2}-1}-t_{l,j}}-\frac{N_{j+1,k_{2}-1}\left(t_{l}\right)}{t_{l,j+k_{2}}-t_{l,j+1}}\right]\\ & \;\times N_{i,k_{1}}\left(t_{c}\right)N_{k,k_{3}}\left(t_{r}\right),\\ \frac{\partial N}{\partial t_{r}} & =\left(k_{3}-1\right)\left[\frac{N_{k,k_{3}-1}\left(t_{r}\right)}{t_{r,k+k_{3}-1}-t_{r,i}}-\frac{N_{k+1,k_{3}-1}\left(t_{r}\right)}{t_{r,k+k_{3}}-t_{r,k+1}}\right]\\ & \;\times N_{j,k_{2}}\left(t_{l}\right)N_{i,k_{1}}\left(t_{r}\right). \end{array}$$

To get the derivatives of B-spline basis functions in *X*, *Y*, *Z* directions, the partial derivative transform of isoparametric principles [10] is used

(14) $$\left[\begin{array}{c} \frac{\partial N}{\partial x}\\ \frac{\partial N}{\partial y}\\ \frac{\partial N}{\partial z} \end{array}\right]=J^{-1}\left[\begin{array}{c} \frac{\partial N}{\partial t_{c}}\\ \frac{\partial N}{\partial t_{l}}\\ \frac{\partial N}{\partial t_{r}} \end{array}\right],$$

where *J* is Jacobian matrix as follows:

(15) $$J=\left[\begin{array}{ccc} \begin{array}{c} \frac{\partial x}{\partial t_{c}}\\ \frac{\partial x}{\partial t_{l}}\\ \frac{\partial x}{\partial t_{r}} \end{array} & \begin{array}{c} \frac{\partial y}{\partial t_{c}}\\ \frac{\partial y}{\partial t_{l}}\\ \frac{\partial y}{\partial t_{r}} \end{array} & \begin{array}{c} \frac{\partial z}{\partial t_{c}}\\ \frac{\partial z}{\partial t_{l}}\\ \frac{\partial z}{\partial t_{r}} \end{array} \end{array}\right].$$

Here, take ∂*x*/∂*t*_*c*_ as an example. *p*_*i*,*j*,*l*_^*x*^ is the control point coordinate in the *X* direction.

(16) $$\frac{\partial x}{\partial t_{c}}=\overset{m}{\underset{i=0}{\sum }}\overset{l}{\underset{j=0}{\sum }}N_{j,k_{2}}\left(t_{l}\right)N_{k,k_{3}}\left(t_{r}\right)\overset{n}{\underset{i=0}{\sum }}N_{i,k_{1}-1}\left(t_{c}\right)\frac{{p^{x}}_{i,j,l}-{p^{x}}_{i-1,j,l}}{t_{c,i+k_{1}-1}-t_{c,i}}.$$

Therefore, the strain can be calculated in this way. By (8), the stress condition can also be calculated.

## 4. Experiments and Results

### 4.1. Construction of Ventricular Models

**Figure 3:**
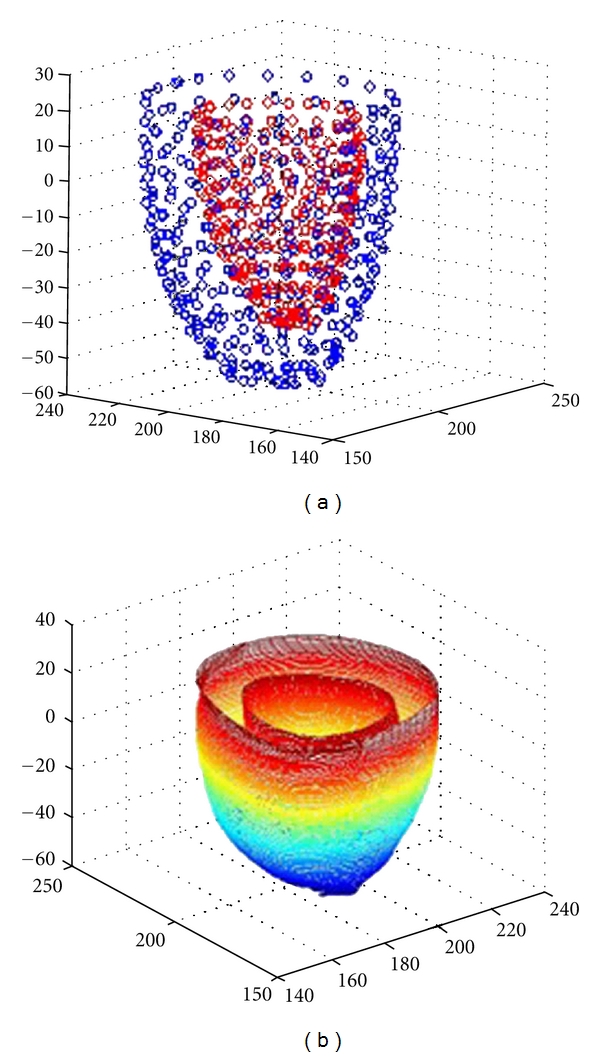
(a) Ventricular point distribution model (b) B-spline surface model.

Both inside and outside data points of ventricular myocardial walls can be obtained from 3D medical images [11, 12].  Figure 3(a) shows the points obtained by model-based segmentation [13].  Figure 3(b) is the corresponding B-spline surface model.

**Figure 4:**
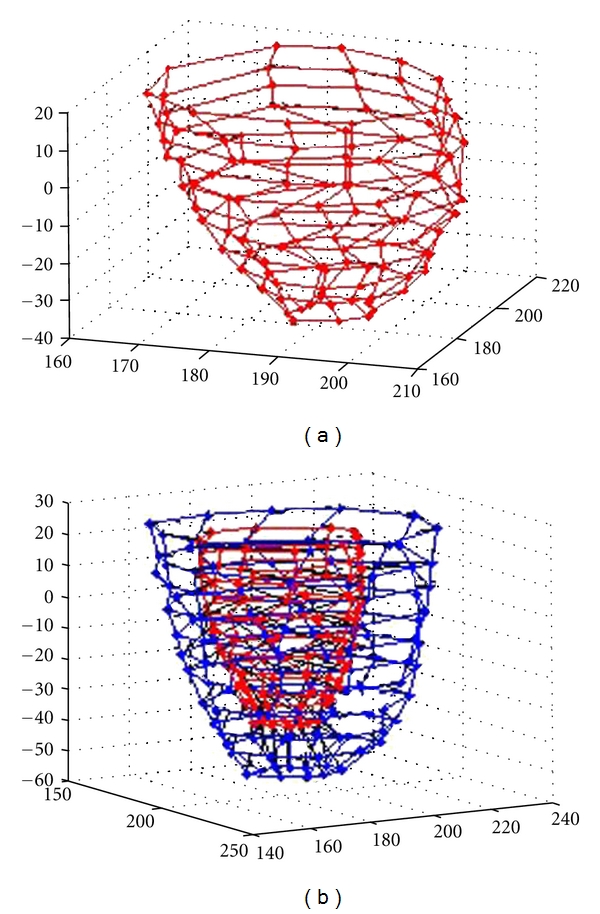
(a) Left ventricular inside wall quadrilateral mesh, (b) hexahedral control mesh.

A sweeping method [14] can be used to obtain the control hexahedral, for example, the one in Figure 4, where Figure 4(a) is the quadrilateral mesh of inside wall, and Figure 4(b) is the hexahedral mesh. While control hexahedral mesh is obtained, the corresponding ventricular B-spline solid model can be reconstructed by (4) and (5).

### 4.2. Stress and Strain

**Figure 5:**
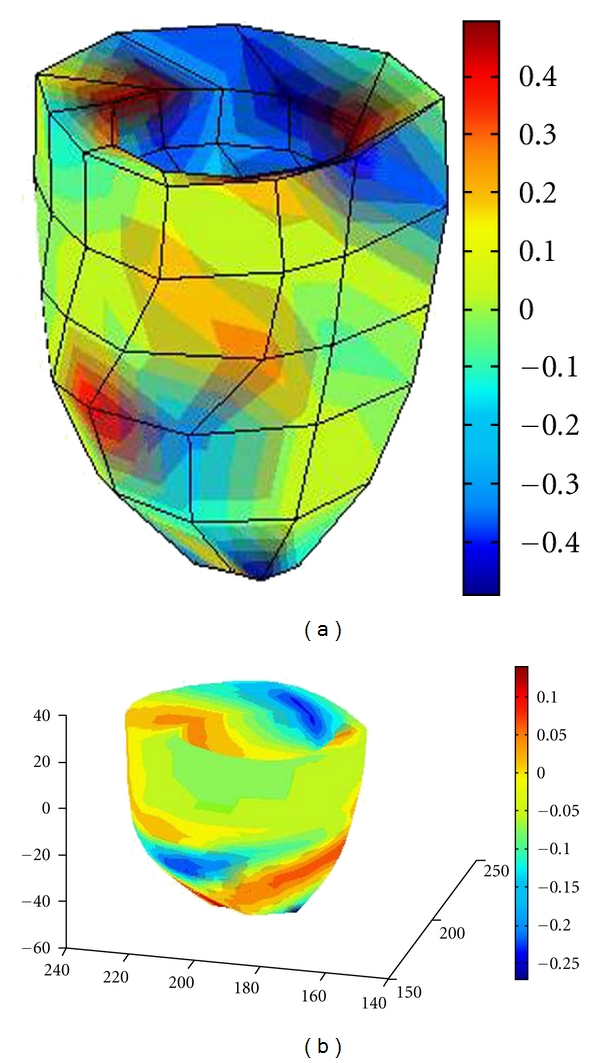
Principal strain distribution in *X* direction: (a) the results by finite element model, (b) result by B-spline solid model.

**Figure 6:**
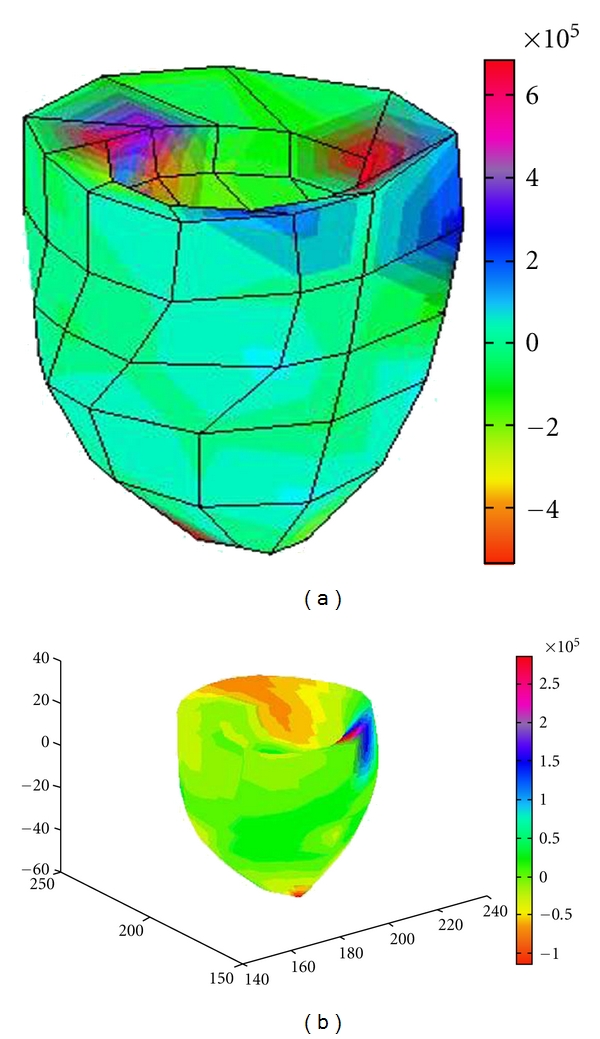
Principal stress distribution in *X* direction: (a) the results by finite element model, (b) result by B-spline solid model.

Stress and strain of a ventricle are calculated based on the steps described in the above sections. Here, for calculation of the stress results, we set myocardial elastic modulus 11 Kpa and Poisson's ratio of 0.49.  Figure 5 shows the principal strain distribution in *X* direction, and Figure 6 shows the principal stress distribution. In these figures, the left one shows the result by the finite element model, and the right shows that by the B-spline solid model. The color represents the change in stress or strain distribution, with the specific reference of the color bar. From the results, it can be seen that the stress and strain of the left ventricular model show overall nonuniform distribution [15, 16], which is consistent with the results by other researchers [17].

## 5. Conclusion

With the situation that traditional finite element methods are difficult for direct use in mechanics analysis which has continuous distribution in space, this paper proposes a solid B-spline model to construct a continuous ventricular mechanical model and applies isogeometric analysis. Stress and strain calculative formulas are derived. The proposed model features continuous, smooth, and inseparable. According to a set of ventricular hexahedral solid B-spline models sampled at different times in a cardiac cycle, strain and stress are determined for medical analysis.
